# The Niagara Meeting

**Published:** 1868-10

**Authors:** 


### EDITORIAL DEPARTMENT.

*The Niagara Meeting.*—Our visit to Niagara Falls in July last, to attend the meeting of the American Dental Association, was a pleasant and profitable one.

We arrived after the stormy period of organization was over, which resulted in the election of Dr. J. Taft as President, who presided at the different meetings in a manner creditable to himself and pleasant to the delegates in attendance.

Our visits from year to year to the meeting of this Association, have convinced us of the necessity existing for the organization of a *Southern Dental Association;* one confined to the late slave holding States. Among the hundreds present each year at the meeting of the Am. Dental Association, but few Southern men are to be found, and an Association which would be confined to the late slave holding States and which would meet in one of these States, would, we have no doubt, be the means of infusing such a spirit in the profession South as to greatly benefit it.

In many of these States there already exist State dental organizations, but one two of which are represented at the meetings of the American Dental Association. Inquiring into the cause of this, we find that the impression has become general among Southern Dentists that a *sectional feeling* governs the action of the majority of the members of the American Dental Association; and on account of such a feeling as this being manifested at every meeting, they decline attending.

This country is large enough for more than one such body as the American Dental Association, and if the organization of another, wholly confined to the Southern States, would be the means of having all these States represented at its meetings, then we are decidedly in favor of such an organization, and at as early a period as is possible.

We should be pleased to hear more on this subject from some of those who are directly interested in the organization of such an Association.

